# The Mucus of *Actinia equina* (Anthozoa, Cnidaria): An Unexplored Resource for Potential Applicative Purposes

**DOI:** 10.3390/md13085276

**Published:** 2015-08-19

**Authors:** Loredana Stabili, Roberto Schirosi, Maria Giovanna Parisi, Stefano Piraino, Matteo Cammarata

**Affiliations:** 1 Institute for Marine Coastal Environment (Unit of Taranto), National Research Council (IAMC-CNR) Via Roma 3, 74100 Taranto, Italy; 2 Department of Biological and Environmental Sciences and Technologies (DiSTeBA), Università del Salento, Via Prov.le Lecce-Monteroni, 73100 Lecce, Italy; E-Mail: stefano.piraino@unisalento.it; 3 Lachifarma s.r.l., S.S.16 Zona Industriale, Zollino (Le) 73010, Italy; E-Mail: robertoschirosi@libero.it; 4 Department of Biological, Chemical and Pharmaceutical Sciences and Technologies (STEBICEF), Università di Palermo, Via Archirafi, 18, 90123 Palermo, Italy; E-Mails: mariagiovanna.parisi@unipa.it (M.G.P.); matteo.cammarata@unipa.it (M.C.); 5 National Interuniversity Consortium for Marine Sciences (CoNISMa), Local Research Unit Lecce, Lecce 73100, Italy

**Keywords:** mucus, *Actinia equina*, antibacterial activity, hemolytic activity, cytotoxicity, tumor cell line K562

## Abstract

The mucus produced by many marine organisms is a complex mixture of proteins and polysaccharides forming a weak watery gel. It is essential for vital processes including locomotion, navigation, structural support, heterotrophic feeding and defence against a multitude of environmental stresses, predators, parasites, and pathogens. In the present study we focused on mucus produced by a benthic cnidarian, the sea anemone *Actinia equina* (Linnaeus, 1758) for preventing burial by excess sedimentation and for protection. We investigated some of the physico-chemical properties of this matrix such as viscosity, osmolarity, electrical conductivity, protein, carbohydrate, and total lipid contents. Some biological activities such as hemolytic, cytotoxic, and antibacterial lysozyme-like activities were also studied. The *A. equina* mucus is mainly composed by water (96.2% ± 0.3%), whereas its dry weight is made of 24.2% ± 1.3% proteins and 7.8% ± 0.2% carbohydrates, with the smallest and largest components referable to lipids (0.9%) and inorganic matter (67.1%). The *A. equina* mucus matrix exhibited hemolytic activity on rabbit erythrocytes, cytotoxic activity against the tumor cell line K562 (human erythromyeloblastoid leukemia) and antibacterial lysozyme-like activity. The findings from this study improve the available information on the mucus composition in invertebrates and have implications for future investigations related to exploitation of *A. equina* and other sea anemones’ mucus as a source of bioactive compounds of high pharmaceutical and biotechnological interest.

## 1. Introduction

To adhere on immersed substrata in their aquatic habitats, many marine organisms, including invertebrates, secrete viscoelastic adhesive gels such as mucus consisting primarily of a network of polysaccharides and proteins entangled to form a weak gel containing more than 95% water [1,2,3,4]. Different from synthetic glue polymers, these bio-molecules are produced in an aqueous environment, therefore including water as a key constituent [5]. This represents a major difference between natural adhesive and synthetic polymers. Mucus is essential to several aquatic organisms for various reasons, e.g., to reduce drag forces, prevent sedimentation, enhance adhesion, limit water loss, and facilitate locomotion [6]. In addition mucus can serve as a “scaffolding” that provides anchorage and protection for egg-laying and a barrier against infection [3]. A mucus layer indeed provides a physical shield [7] and a slippery coating that prevents bacteria and debris from accumulating on the body surface [8], with a number of defence mechanisms [9,10,11,12,13]. Many marine invertebrates are sessile, *i.e.*, steadily attached to the sea bottom or with low locomotion ability, thus vulnerable either to predation and threat from a rich surrounding microbiota with pathogenic potential. Besides mechanical protection, the mucus of many invertebrates contains specific compounds to make the animal poisonous, distasteful or irritating, or a combination of these features [14]. Also, it is not surprising that these invertebrates developed an innate immune system producing a considerable number of defence molecules such as lytic compounds [15], bioactive antimicrobials [16,17,18], toxins, and carbohydrate antiadhesives [19]. Lectin-like molecules able to agglutinate red blood cells were characterized from mucus of the gastropod snail *Helix aspersa*, whose agglutinating activity was inhibited by d-Ga1NAc [20]. In addition, the potential to reduce the bacterial adhesion was demonstrated from mucus glycoproteins of the starfish *Marthasterias glacialis* [21], together with an antibacterial lysozyme-like activity [22], also observed in the annelid polychaetes *Sabella spallanzanii* [10,23] and *Myxicola infundibulum* [12].

As suggested by Calow [24], mucus could be made more or less susceptible to microbial attack. Some invertebrates could lace their mucus with antibiotic molecules when it is more advantageous for them to inhibit bacterial attack; in those cases, the mucus contains less proteins and does not promote bacterial growth. By contrast, some invertebrates, including corals [25], may release mucus with high content of proteins rapidly used by microbes. Due to their high turnover rates and their physiological diversity, microbes are likely to react quickly to released protein-rich mucus. Bacteria indeed possess a wide range of exo-enzymes potentially capable of degrading mucoid polymers, boosting the development of a mucus-specific microbiome. These microbes may transform mucus-derived (dissolved and particulate) organic matter into living biomass, *i.e.*, forming the so-called “microbial loop” trophic pathway [26], where mucus can be the scaffolding matrix eventually supporting a mucus-based food web [27,28,29].

Most cnidarians, including both medusozoans and anthozoans, are capable of secreting a mucus-based surface layer essential for a number of processes such as feeding, protection against pathogens, desiccation, and a number of environmental stresses. Mucus production may account for as much as 40% of the net daily fixed carbon in the coral *Acropora acuminata* [30]. Other uses that should be considered are protection from aggression and as an offensive weapon. The coral *Lobactis* (*Fungia*) *scutaria* in response to contact with other corals or rough human handling secretes mucus containing cytotoxic molecules to other corals. A highly active cytolysin as well as aliphatic-antibiotic compounds have been isolated from the mucus secretion of the sea anemone *Heteractis magnica* [31]. In spite of the multitude of ecological and physiological roles played by the cnidarian mucus, relatively little is known about the link between biochemical structures and functions. In the present study we focused on the mucus of the intertidal sea anemone *Actinia equina* produced as mechanical protection against excess sedimentation or desiccation as well as barrier against microbial attacks. Tissue extracts of *A.equina* has been long investigated for their peptide and protein toxins. Besides at least five isoforms of pore-forming cytolysins (equinatoxins) of proteinic nature, tissues of *A. equina* also contain several peptide toxins (Ae I, Ae K, acrorhagin I and II) isolated from different body portions [32,33]. Here, we investigated some of the physico-chemical properties of the secreted mucus of *A. equina* such as viscosity, osmolarity, electrical conductivity, protein, carbohydrate, and total lipid contents. Some biological activities, such as the hemolytic, cytotoxic, and antibacterial lysozyme-like activities were also investigated to highlight the potential of sea anemone mucus as a source of bioactive compounds of interest for biotechnological applications.

## 2. Results

### 2.1. Mucus Viscosity, Osmolarity, and Electrical Conductivity

Adult specimens of *A. equina* were employed for both the study of the physical and chemical properties of the mucus and the determination of its biological activities. The mean viscosity of *A. equina* mucus was 2.1 ± 0.02 cPs in respect to the 1 cPs viscosity of water measured at 20 °C (Table 1). The mean osmolarity value of the cnidarian mucus was 1205 ± 10 mOsmol/L, similar to seawater (1152 ± 25 mOsmol/L). The mean electrical conductivity of mucus was 124 ± 4 mS·cm^−1^ whilst the electrical conductivity of the seawater is 35 mS·cm^−1^.

**Table 1:** Main physico-chemical characteristics of *Actinia equina* mucus.

Physico-Chemical Feature	Mean ± SD
Inorganic matter (%)	67.1 ± 2.3
Organic matter (%)	32.9 ± 0.2
Viscosity 20 °C (cps)	2.1 ± 0.02
Osmolarity (mOsmol/L)	1205 ± 10
Conductivity (mS·cm^−1^)	124 ± 4.0

### 2.2. Water and Inorganic Content

**Figure 1:**
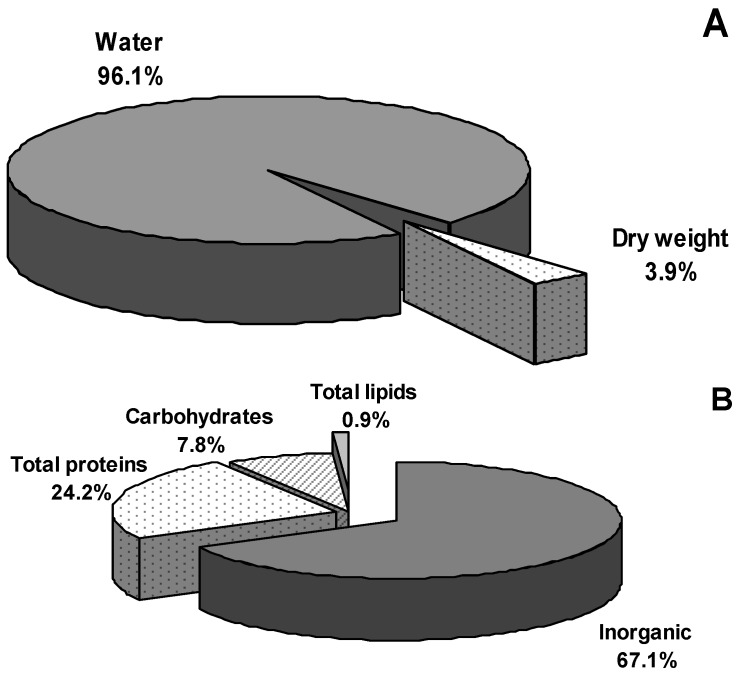
*Actinia equina* mucus composition: (**A**) water content and dried weight; (**B**) organic and inorganic residuals.

The water content of *A. equina* mucus was 96.1% ± 0.5% (Figure 1A). After dehydration, inorganic salts represented the main part (67.1% ± 2.3%) of the mucus dry weight (DW) (Figure 1B). Mean percentages of the elements are listed in Table 2: In all samples, Cl and Na were abundant whereas C, Mg and K represented only 2.8%–2.1% of the inorganic content.

**Table 2:** Elements detected in mucus sample of *Actinia equine.*

Element	Content (%)
Cl	44.48 ± 0.12
Na	13.38 ± 0.11
Mg	2.41 ± 0.03
H	1.53 ± 0.10
K	2.11 ± 0.02
Ca	0.71 ± 0.02
C	2.13 ± 0.02
N	0.45 ± 0.02
Zn	0.06 ± 0.005
Cu	absent
Fe	absent
P	absent
Se	absent
Sn	absent

### 2.3. Protein, Carbohydrate, and Lipids Concentration

**Figure 2:**
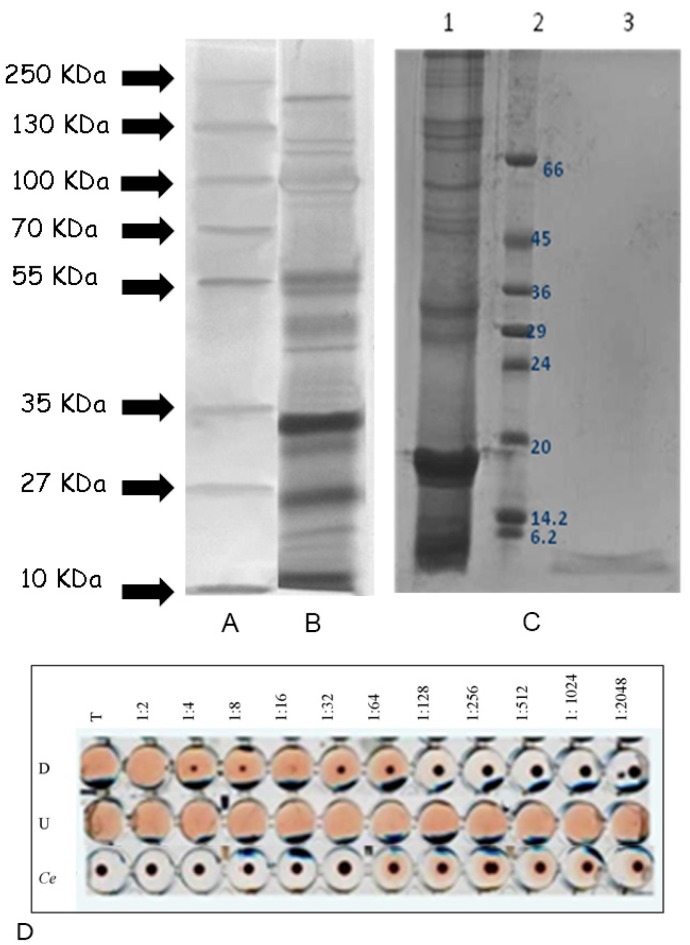
SDS-PAGE analysis of *Actinia equina* mucus. Panel **A**: Molecular weight standards furnished by Fermentas. Molecular weights (kDa) of standard proteins are on left; Panel **B**: *A. equina* total mucus; (**C**) *Actinia equina* different molecular weight fractions from total mucus extract obtained by membrane filtration system (pore size: 10 kDa). SDS-PAGE 15% acrylamide gel stained with Coomassie Blue R-250. Lane 1: Fraction >10 kDa named “U” (Upper), Lane 2: Standard Low sigma, Lane 3: Fraction <10 kDa. Named “D” (Lower); (**D**) Micro plate lysis assay carried out against Rabbit erythrocytes (RRBCs) in TBS buffer. Hemolysis is evidenced by free hemoglobin, when the erythrocytes are not lysed a central pellet of erythrocytes is visible on the well center. Lower fraction (**D**) showing lysis until dilution of 1:64, Upper fraction (U) showing lysis until dilution of 1:2048, Control experiment (Ce) with RRBCs and buffer.

The organic residual of *A. equina* mucus DW was composed of proteins (24.2% ± 1.3%), carbohydrates (7.8% ± 0.2%) and lipids (0.9% ± 0.02%) (Figure 1B) with protein/glucose ratio equals to 3.2. The electrophoretic analysis revealed at least fourteen major protein bands, ranging from 12 to 200 kDa (Figure 2B).

### 2.4. Lysozyme Like Activity

**Figure 3:**
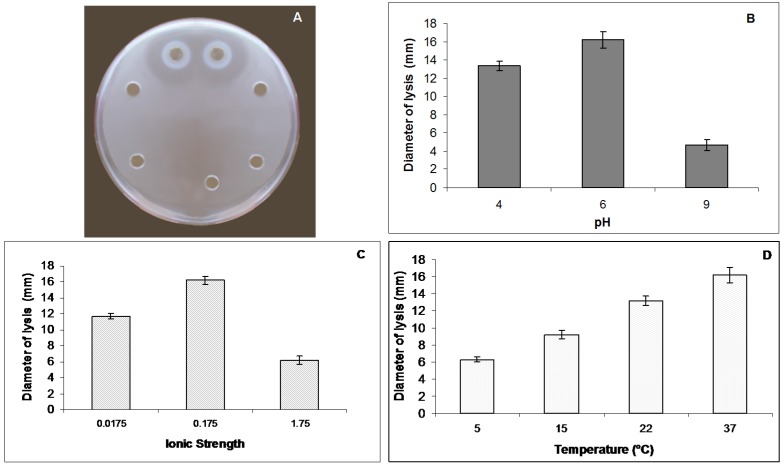
Lysozyme-like activity of *Actinia equina* mucus. (**A**) Standard assay on Petri dish inoculated with *Micrococcus lysodeikticus* cell walls to detect the lysozyme-like activity of *A. equina* mucus; (**B**) Effect of the pH on the lysozyme-like activity of *A. equina* mucus. Columns are mean values (*n* = 20) (vertical bars ± Standard Deviation); (**C**) Effect of the ionic strength on the lysozyme-like activity of mucus. Columns are mean values (*n* = 20) (vertical bars ± Standard Deviation); (**D**) Effect of the incubation temperature on the lysozyme-like activity of mucus. Columns are mean values (*n* = 20) (vertical bars ± Standard Deviation).

Mucus of *A. equina* showed a natural lysozyme like activity (Figure 3A). This activity was strictly affected by pH (Figure 3B) and ionic strength (I) (Figure 3C) of the sample and of the reaction medium. The maximum diameter of lysis was reported at pH 6.0. The lytic activity increased after dialysis of the mucus at pH 6.0 and I = 0.175. The largest diameters of lysis were recorded at 37 °C (Figure 3D). By the standard assay on Petri dishes the maximum diameter of lysis (16.2 ± 0.5 mm corresponding to 2.21 mg/mL of hen egg-white lysozyme) was reported at I = 0.175, pH 6.0 and incubation temperature of 37 °C.

### 2.5. Hemolytic Activity

Mucus of *A. equina* with a protein concentration of 0.8 mg/mL exerted a hemolytic effect after incubation at 37 °C against rabbit and sheep erythrocytes with a lysis titer of 1:526 and 1:1048, respectively.

### 2.6. Cytotoxic Activity

**Figure 4:**
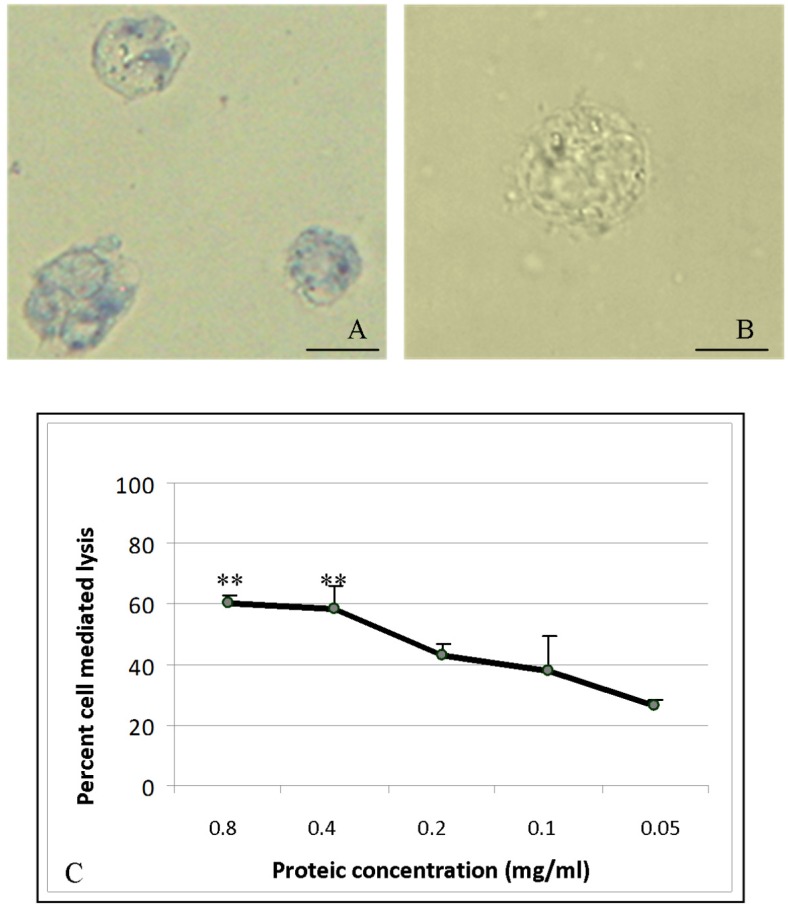
(**A**) Light Microscopic observation of Human erythromyeloblastoid leukemia (K562) cells treated with *A. equine* mucus crude extract. The target cell lysis was also determined by trypan blue exclusion test. Bar: 25 μm; (**B**) Control cell observed in the absence of mucus Bar. 25 μm; (**C**) Colorimetric assay of *A. equina* mucus extract on human chronic myelogenous leukemia cells K562 (Cytotoxic detection Kit. Boehringer Mannheim, Mannheim, Germany). Lactate dehydrogenase release into the supernatant was used to calculate the percentage of target cell lysis.

The trypan blue dye exclusion test was used to determine the number of viable cells present in a cell suspension incubated with *A. equina* mucus sample. Human erythromyeloid leukemia-derived (K562) treated cells were damaged by mucus compounds (Figure 4A). Control cells (without mucus incubation) show intact cell membranes and do not incorporate trypan blue (Figure 4B).

The mucus of *A. equina* exhibits direct cytotoxic activity on K562 target cells (Figure 4C). Lactate dehydrogenase release into the supernatant of cells was used to calculate the percentage of target cell lysis. At the mucus protein concentration of 0.8 mg/mL and 0.4 mg/mL, the percentage of lysis was found significantly higher than control cells and quantified respectively equal to 62% and 58% of total target cells in suspension.

### 2.7. Fractionation of Actinia Equina Mucus

The system of separation by centrifugation through Nanosep devices membrane has allowed to obtain two fractions starting from the sample of mucus (1.2 ± 0.3 mg/mL). Molecules larger than the membrane pores of 10 kDa were retained at the surface of the membrane and concentrated during the ultrafiltration process. This component was defined “U” (upper) with a concentration of 1.5 ± 0.2 mg/mL, while the fraction with molecular weight below 10 kDa was named “D” (lower) (0.256 ± 0.022 mg**/**mL). The SDS electrophoresis analysis of the two mucus components showed a major component to occur in the D fraction, with an apparent mass less than 6 kDa (Figure 2C).

In microplate the isolated fractions showed a different lytic activity toward rabbit erythrocytes (Figure 2D). The lysis capacity was identified until 1:2048 dilution of the sample in the U fraction, and till dilution of 1:64 in the D fraction.

**Figure 5:**
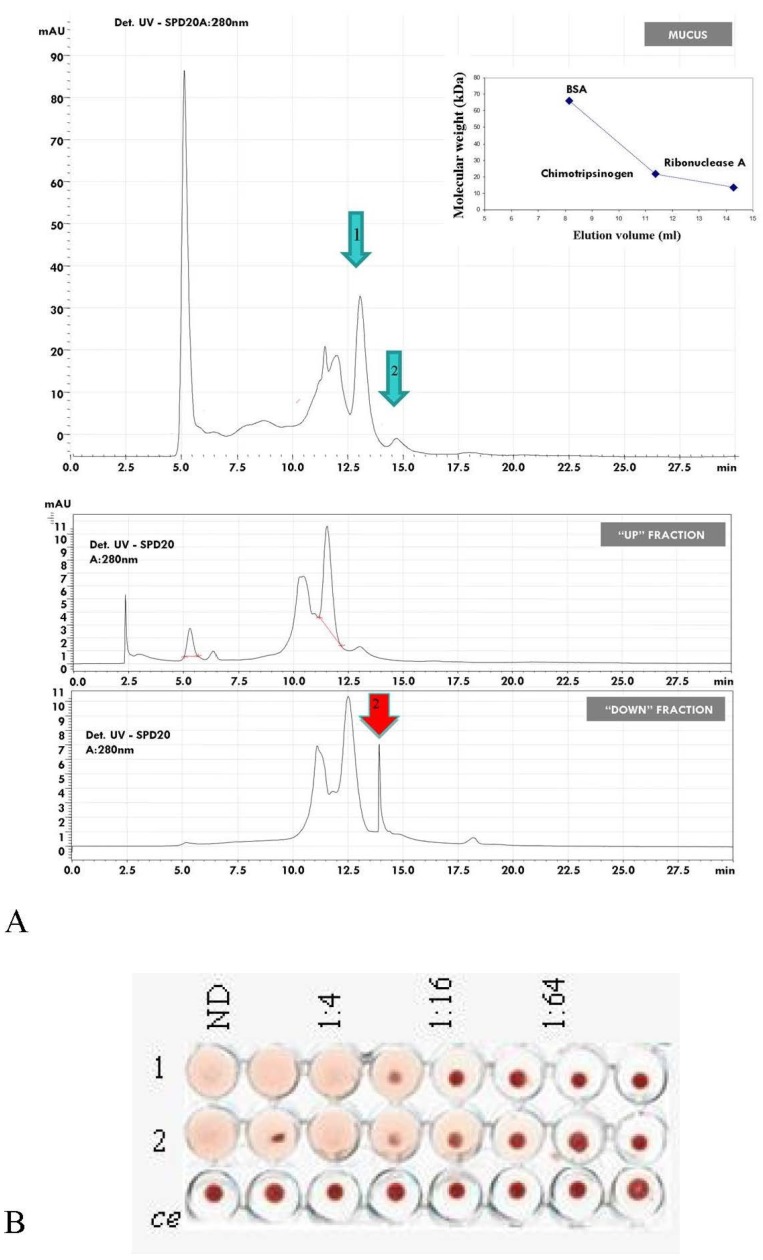
(**A**) High performance liquid chromatography separation of *A. equina* mucus components. The first plot shows profile of HPLC analysis of the crude mucus extract. Green arrows 1 and 2 indicate the isolated peaks at 12.5 and 14.5 min. Insert shows HPLC profiles of bovine serum albumin (BSA-66 kDa), chimotrypsinogen (25 kDa) and ribonuclease (13.7 kDa) used as standards separated on a molecular weight exclusion column BioSuite 250 (10 microns; Waters, Milford, CT, USA). The second plot shows the purification profiles of the high molecular weight fraction (u = upper) and low molecular weight fraction (d = lower) previously separated via centrifugation system on 10 kDa membrane. Red arrow indicate peak 2 detected at 14.5 min post HPLC start running; (**B**) Lytic activity detected in microplate toward rabbit erythrocytes of peaks 1 and 2 (Ce: Control experiment).

### 2.8. HPLC Separation of Mucus Components

Profiles of high pressure liquid chromatography (HPLC) on a column of size exclusion chromatography (BioSuite 250, 10 μm SEC, 7.5 mm × 300 mm) revealed the separation plot obtained from the mucus sample and the U fraction to be similar (Figure 5A). Two peaks are detected at 12.5 min (1) and 14.5 min (2). The first one contains proteins with a high molecular weight while the second peak includes molecules of lower dimensions. The profile of purification of the D fraction resulted enriched in the second component of low molecular weight. All the HPLC fractions of 1 mL/min were collected and subsequently analyzed to assess the hemolytic activity. The results of the assays (Figure 5B) showed the presence of hemolytic activity toward rabbit erythrocytes up to 1:16 for the peak 1 and 1:32 for the peak 2. Thus, different components within the sample showed similar hemolytic activity. Comparing the elution volume of standards used before purification of samples, the fraction 1 contains mainly a 20 kDa electrophoretic band while the peak 2 includes the 6 kDa component. The nature of equinatoxins-related activity of the 20 kDa fraction was suggested by inhibition experiments using bovine sphingomyelin (data not shown). One of the hallmarks of actinoporins is they efficiently make pores in lipid membranes containing this lipid. Thus, the interaction between erythrocytes membrane lipids and lysins was evaluated by inhibition experiments carried out using rabbit erythrocytes.

## 3. Discussion

Underwater attachment will undoubtedly have many technological applications including the design of water-resistant adhesives, sealants, and biomedical coatings and the development of new antifouling strategies [34]. Nonetheless a considerable dearth of information still remains regarding the biochemical composition of marine adhesives and the link between biochemical structure and function. Hitherto, studies on adhesives from invertebrates mainly concerned the characterization of permanent secretions from mussels and barnacles [5,35]. In comparison, non-permanent adhesives, (more hydrated than permanent ones and consisting of a mixture of proteins and polysaccharides) received so far much less attention [4,36,37,38,39]. The present paper represents a preliminary contribution on this topic since we provide novel data on the physico-chemical and biological properties of the sea anemone *A. equina* mucus. We analyzed some rheological properties of mucus such as osmolarity and viscosity since they are believed to be critical in fulfilling specific biological functions and are intimately related to the chemical composition [23]. Compared to mucus from typical marine sources [40] with a water content ranging 96%–98% of the wet weight, the mucus of *A. equina* has water content up of 96.2% ± 0.3%. The high percentage of inorganic material (about 67.1%) presumably results from dried salts left over when the seawater in a gel evaporates as already suggested for limpets and periwinkles mucus by Smith *et al.* [4] and Smith and Morin [40] which observed a similar proportion of inorganic material. The mucus of the studied cnidarian is mostly composed of proteins, representing the most conspicuous organic component (24% of total mucus dry matter, 73% of dry organic matter residual), and carbohydrates (7.8% of total mucus dry matter, near 24% of dry organic matter residual). Similar values were recorded for the mucus of limpets [4] as well as for the mucus of the annelid polychaete *Sabella spallanzanii* [10]. From studies on the biochemical composition of adhesive footprints of the sea star *Asterias rubens* [41,42] the amount of protein and carbohydrate (20% and 8% respectively) resulted similar to those recorded in *A. equina* mucus. As observed in the mucus of *A. equina*, the co-occurrence of proteins and carbohydrates seems to be a common trait among non-permanent adhesives of marine invertebrates, from cnidarians to deuterostomes [37] and protostomes [40,43]. These protein-carbohydrate complexes typically form highly hydrated adhesives with viscoelastic properties [42,43]. The mucus of *A. equina* corresponds to this kind of adhesives also on account of the obtained values of viscosity and osmolarity. This matrix indeed exhibits a low viscosity (2.1 ± 0.02 cps) and its osmolarity is of the same order of magnitude as the seawater such that mucus achieves near ionic equilibrium with the surrounding medium.

A noteworthy result of the present paper is the protein pattern of the *A. equina* mucus: from the electrophoretic analysis a complex of at least fourteen major proteins ranging from 12 to 200 kDa was highlighted. This is in agreement with the general multi-protein nature of other marine invertebrate adhesives. Indeed, in the sea urchin *Paracentrotus lividus* footprint material SDS-PAGE analysis revealed that the soluble fraction contains about 13 protein bands with molecular masses ranging from 10 to 200 kDa [39]. Moreover, in the mucus of the polychaete *Sabella spallanzanii* the electrophoretic analysis revealed at least 10 major protein bands, with molecular weights ranging from 16 to 90 kDa, and six minor components, with molecular weights ranging from 14 to 116 kDa [23]. In non-permanent adhesives, multi-protein complexes have been also evidenced in sea cucumbers and limpets [34,43]. The lysozyme activity recorded in mucus of *A. equina* mucus can be ascribed to one the fourteen major protein bands evidenced by electrophoretic analysis. Interestingly, one of the “known proteins in the databases” described in a marine adhesive is a homolog of lysozyme in barnacle cement [44]. Lysozyme represents the best characterized enzyme involved in self-defence from bacteria [45]. This enzyme is a glycoside hydrolase and dissolves certain bacteria by hydrolyzing the glycosidic β1-4 bonds between *N*-acetylglucosamine and *N*-acetylmuramic acid of bacterial cell walls. A bacterial cell devoid of a wall usually bursts because of the high osmotic pressure inside the cell. The constitutive levels of lysozyme protect the organism from bacteria living in the same environment and control its natural symbiotic flora. Lysozyme-like proteins have also already been found in other cnidarians, including some species of sea anemones [46,47]. In the present work it a lysozyme-like activity was also highlighted in the mucus of *A. equina*. This lysozyme had a maximum of activity when the pH of the reaction medium and sample was 6 and the ionic strength 0.175, as previously reported for other lysozymes [48,49,50,51,52].

We also showed hemolytic activity of *A. equina* mucus extract toward RRBC target cells. After purification by membrane separation system and HPLC, different components exhibiting hemolytic activity have been found. Among the mucus components of high molecular weight, fraction “up”, the hemolytic activity resides in a 20 kDa protein corresponding to Equinatoxin, as demonstrated by the experiment of hemolysis carried out on peaks isolated by HPLC. Results of hemolytic assays showed also an active fraction with a lowest molecular weight, of approximately 6 kDa in SDS. Both these lytic fractions are inhibited by sphingomyelin. This suggests that the mucus of *A. equina* could contain actinoporin-like molecules, known to be specifically inhibited by sphingomyelin [53] which may have an interaction with erythrocyte membrane permeability, leading to lysis. Actinoporins belong to the unique family of the α-pore-forming toxins (PFTs) due to their ability to hold the membrane phospholipids domains of the host organism forming cation selective pores [54]. Interestingly, the mucus of *Heteractis magnifica* showed a strong hemolytic activity toward fish erythrocytes, and exerted an antibacterial activity towards pathogenic bacterial strains. The presence in *A. equina* mucus of a hemolytic, cytotoxic activity and an antibacterial activity suggests that this matrix may provide a defensive tool for the cnidarian from microbial attacks serving as substrate into which the humoral substances are released. The role of mucus as a defence against potentially pathogenic microorganisms has been already demonstrated for the mucus of other marine invertebrates including corals [3,9]. The antibacterial functions of coral mucus are particularly well documented in the soft corals. Slattery *et al.* [55] demonstrated anti-microbial and anti-fouling activity in Antarctic soft corals also and suggested that, although mucus secretion in these species was low, it was likely to be important in preventing bacterial attachment to the coral surface.

The results obtained in the present study not only improve the available information on the mucus composition in invertebrates, but also have implications for future studies aimed to the employment of *A. equina* mucus as source of compounds with antimicrobial lysozyme-like and antitumor activity of pharmaceutical and biotechnological interest. As regards pharmaceuticals, the ongoing explosion of antibiotic-resistant infections due to new opportunistic pathogen multidrug-resistant microbes continues to plague global health care. This clearly highlights the need for new antibacterial agents with fundamentally different modes of action than that of traditional antibiotics. The enormous demand has triggered worldwide efforts in developing novel antibacterial alternatives. Bacterial cell wall hydrolases (BCWH) are among the most promising candidates and lysozyme was recently chosen as a model protein. For the first time, this led a great opportunity for potential use of lysozyme in drug systems as a new antimicrobial agent [56,57]. A possible application of lysozyme, which is attracting considerable interest, is the use of this molecule in veterinary work and in aquaculture facilities in particular. The emergence of microbial diseases in aquaculture industries is of major concern implying serious financial loss. Therefore, *A. equina* mucus appears as a promising and valuable alternative source of lysozyme for drug development and the marine origin of this lysozyme represents an added value. Last but not least, indeed, the lysozyme produced by *A. equina* mucus is salt-stable and this feature makes it more suitable to be used to control fish or shellfish pathogens in mariculture in the case of antibiotic efficacy reduction due to high-salt conditions.

The utilization of *A. equina* mucus to extract bioactive substances of pharmaceutical interest is encouraged also with the evidence of the cytotoxic activity against the tumor cell line K562. In *A. equina* the first indication about cytotoxicity of its venom due to equinatoxin action was elucidated by dye exclusion test on Ehrlich carcinoma and L1210 leukemia inoculated in mice [58]. In another study, crude extracts from nematocyst and surrounding tissues of the sea-anemone *A. equina* were tested on V79 fibroblasts [59]. Moreover Isoform II of Equinatoxin (Eq. II) *showed* cytotoxic capability against human glioblastoma U87 and A172 cell lines [60]. Eq. II was found to affect the survival of U87 glioblastoma cells by a necrosis-like action and increasing lactate dehydrogenase (LDH) release [61]. On account of our results it seems that in addition to Eq. II a low molecular component, responsible for the toxicity to K562 tumor cells, is present in the mucus. This finding demonstrates that not only nematocysts or the granulocytes of *A equina* produce and release cytotoxins [62] but also the matrix outside of the body which, releasing toxic substances, is involved in defense mechanisms.

Finally, the antibacterial and cytotoxic activity of *A. equina* mucus could be employed to avoid the settlement of bacteria, which is the primary colonizing process in marine biofouling development. Alternative marine technologies employing biogenic compounds that function as natural anti-settlement agents are sought taking into account that some compounds such as TBT, copper [63], and organic biocides [64] used as antifouling agents in paints have been banned after 2008 [65,66,67]. Recently we have also purified new thermo-stable proteases and antimicrobial peptides from the body and tentacle of *A. equina* and *Anemonia sulcata* which were applied for biocleaning or controlling microbial growth on heritage objects [68]. In particular, the protease-containing fraction was tested for the hydrolysis of protein layers on old paintings. The cleaning protocol including sea anemone proteases offered a novel selective procedure preventing damage to the original materials constituting the heritage object. The fraction containing the antimicrobial peptide was used to control fungal growth during the restoration of the painting [68]. Bioactive molecules extracted from sea anemones’ mucus are currently under investigation.

## 4. Experimental Section

### 4.1. Animals and Samples Preparation

Adult specimens of *Actinia equina* were collected at Porto Cesareo (Lecce, Italy, 40.25 N, 17.9 E) using SCUBA equipment.

About 100 adult specimens of *A. equina* were collected and transferred to the laboratory. Here the sea anemones were washed with filtered (0.2 µm) sterile sea water and kept for 30 min in a Petri dish in order to stimulate the secretion of the mucus for both the study of its physico-chemical properties and the determination of its biological activities such as hemolytic, cytotoxic and antibacterial, lysozyme-like activities. Within the secreted mucus, we checked for trapped material by microscopic observations, whilst we excluded any contamination of other excretion products by pH measurements. Secreted mucus was collected and centrifuged at 12,000× *g* for 30 min at 4 °C. A previous work [10] showed that the protein content of the mucus varies between individuals. To avoid the introduction of this variable, in the present work the mucus of the whole group of 100 individuals was pooled into five samples (each pool collected from 20 sea anemones) which were stored at −80 °C until use.

### 4.2. Mucus Viscosity, Osmolarity, Electrical Conductivity and Water Content

Mucus viscosity was measured at 200 rpm in 1 mL aliquots with a cone-plate viscometer (cone angle of 1.565°, model LVT-C/P 42, Brookfield Engineering Laboratories, Middleboro, MA, USA) connected to a circulating water bath (Thermoline, Wetherill Park, Sydney, Australia) set at 17 ± 0.1 °C. Due to differences in temperature and equipment used between studies, comparison of viscosity data can be difficult without reference to a common, known viscosity. Thus, we documented the relative viscosity of mucus with respect to the viscosity of water, similar to Rosen and Cornford [69] and Cone [70]. The viscosity of water is 1 cP at 20 °C and it is only slightly dependent on temperature [71].

Osmolarity was measured using a VAPRO vapour pressure osmometer (model 5520, WESCOR, Logan, UT, USA), all measurements being carried out in triplicate. Electrical conductivity was measured using a GLP 31 conductimeter (Crison, Barcelona, Spain).

For water content measurement, the wet weights of mucus of 15 samples (three replicates for each of the five groups of 20 individuals each) were measured on an analytical balance. They were then dehydrated in a SpeedVac, and their dry weight (DW) was measured.

### 4.3. Determination of the Inorganic Composition

The inorganic composition was determined for each sample after lyophilization of sample solution at 52 °C and 0.061 mbar using a LIO 5P CINQUEPASCAL freeze-dryer.

C, H, and N analyses were performed using a 1106 Carlo Erba elemental analyzer, while an AA-6200 Shimadzu atomic absorption flame emission spectrophotometer was used for the determination of Fe, Ca, Mg, Zn, Cu, K, Na. A P/N 206-17143 Shimadzu hydride vapor generator was coupled to the atomic absorption spectrophotometer in order to analyze the Sn and Se content. In general, each sample was mineralized to oxidize the organic fraction. To this end a weighted sample of the mucus (*ca.* 10 mg) was treated with HNO_3_ (1 mL) and H_2_SO_4_ 96% *w*/*w* (2.5 mL) at high temperature until no more fumes were released. The residue was treated again with the acids two more times. The final liquid residue was dissolved in water to give a 100 mL solution. For each element a calibration curve was obtained by using standard solutions. The quantitative analysis of phosphorous was performed using an UV-1601 Shimadzu spectrophotometer according to the method reported in the literature [72,73]. A 785 DMP Metrohm Titrino was used for the quantitative determination of the inorganic chloride using a potentiometric determination.

### 4.4. Lipid, Protein, and Carbohydrate Concentration

Total lipids from each mucus sample were extracted according to the method of Folch *et al.* [74]. The mucus was homogenized with chloroform/methanol (2:1) to a final volume 20 times the volume of the mucus sample. After centrifugation and siphoning of the upper phase, the lower chloroform phase contained the lipids. Total lipid content was determined by the colorimetric enzymatic method [75] using commercial kit (FAR, Verona, Italy).

The protein concentration of each mucus sample was measured using the Bradford assay [76] with bovine serum albumin (BIO-RAD, Hercules, CA, USA) as standard.

The carbohydrate concentration of the mucus was assayed using the method described by Dubois *et al.* [77] and Kennedy and Pagliuca [78]. The assay was calibrated with known amounts of d-glucose.

### 4.5. Electrophoresis

Mucus samples were analyzed by SDS-polyacrylamide gel electrophoresis (SDS-PAGE). They were run on discontinuous gels, based on the method of Laemmli [79] and the detailed protocols of Hames [80]. The gels contained 10% of acrylamide, and were 8 cm × 9 cm by 1.0 mm thick. The migration buffer consisted of 25 mM Tris, 192 mM glycine, pH 8.5. After migration, gels were stained using Silver Stain kit (Sigma, Saint Louis, MO, USA). Molecular standards (PageRuler™ Prestained Protein Ladder range 10–250 kDa, Fermentas, Waltham, Massachusetts, USA) consisted in a mixture of eight recombinant, highly purified, coloured proteins with apparent molecular weights of 10 to 250 kDa.

### 4.6. Lysozyme-Like Activity

To detect lysozyme activity, inoculated Petri dishes were used as standard assay, 700 μL of 5 mg/mL of dried *Micrococcus luteus* cell walls (Sigma, Saint Louis, MO, USA ) were diluted in 7 mL of 0.05 M PB-agarose (1.2%, pH 5.0) then spread on a Petri dish. Four wells of 6.3 mm diameters were sunk in agarose gel and each filled with 30 μL of mucus. The diameter of the cleared zone of the four replicates was recorded after overnight incubation at 37 °C and compared with those of reference samples containing known amounts of standard hen-egg-white lysozyme (Merck, Darmstadt, Germany). The effects of pH, ionic strength (I), and temperature were examined. The pH effect was tested by dialyzing the mucus in PB 0.05 M, ionic strength, I = 0.175, adjusted at pH 4, 5, 6, 7, 8 and by dissolving agarose in PB at the same I- and pH-values. The ionic strength effect was tested in PB 0.05 M (pH 6.0), adjusted at I = 0.0175, 0.175, 1.75. Agarose was dissolved in PB at the same I-values. The temperature effect was tested with incubations of samples (in PB, at pH 6.0, and I = 0.175) at 5, 15, 22, and 37 °C.

### 4.7. Hemolytic Activity

The rabbit erythrocytes (RRBCs) obtained by Istituto Zooprofilattico della Sicilia in Alsever solution (0.42% NaCl; 0.08% sodium citrate dihydrate, citric acid monohydrate 0.045%, 2.05% d-glucose pH 7.2) were washed three times with erythrocytes-Phosphate-buffered saline (PBSE) (KH_2_PO_4_ 6 mM; Na_2_HPO_4_ 0.11 M; NaCl 30 mM; pH 7.4) and centrifuged at 1800 rpm for 10 min at 4 °C. Suspensions of RRBCs (2.5% in Tris buffer: Tris HCl 0.05 M, 0.15 M NaCl pH 8) were used to test the lysis of RRBCs by mucus. For the microplate assay 25 μL of mucus or serial (two-fold) dilution were mixed with an equal volume of the RRBCs suspension.in 96-well round-bottom microtiter plates (Nunc, Roskilde, Denmark). After 1 h incubation at 37 °C the lytic activity was recorded as the reciprocal of the highest dilution showing complete RRBCs lysis.

For the quantitative hemoglobin release evaluation, one hundred microlitres of mucus in triplicate were mixed with 100 µL RRBCs suspension in glass tubes with U bottom, incubated for 60 min at 37 °C and then centrifuged for 5 min at 1500× *g*. One mL of Tris buffer was added to the supernatant in order to obtain an adequate amount of sample for spectrophotometric evaluation (541 nm) of the hemoglobin content. The degree of hemolysis was calculated by: [(absorbance of sample − absorbance of control)/absorbance of total hemolysis] × 100. Total hemolysis (100%) was achieved by adding 100 µL of distilled water to the same volume of RRBCs suspension.

Control erythrocyte suspensions were also prepared in the same medium and incubated as reaction mixtures: spontaneous hemoglobin release never exceeded 5% of the total release. For each experiment three samples were assayed.

### 4.8. Cytoxicity Assay against the Tumor Cell Line K562

The human erythromyeloid leukemia-derived cell line K562 were kindly provided by Dr. Domenico Schillaci (STEBICEF, University of Palermo) and was maintained for short time in RPMI 1640 medium (Gibco, Grand Island, NY, USA) supplemented with 10% heat inactivated fetal calf serum (Flow Laboratories, Irvine, Scotland), gentamycin, streptomycin, and Hepes buffer (Boehringer Mannheim, Mannheim, Germany).

Cytotoxicity effect of mucus against tumor cell lines was performed using a cytotoxic detection Kit (Boehringer Mannheim, Mannheim, Germany) based on determination of lactate dehydrogenase (LDH) activity released from lysed target cells [81]. The target cells were washed and suspended in PBS supplemented with 1% bovine serum albumin (PBS-BSA, 370 mOsm kg^−1^) at a concentration of 10^5^ cells mL^−1^. All tests were performed in triplicate with 10^4^ target cells well^−1^ in V-shaped microplates (Nunc, Roskilde, Denmark) in a total volume of 200 µL. Plates were centrifuged for 1 min at 100× *g* and incubated for 2 h at 18 °C. The plates were then centrifuged for 5 min at 400× *g*, and the release of LDH from lysed cells in 100 µL of supernatant from each well was determined by reading the absorbance at 490 nm in a microplate reader (Uniskan I, Labsystems, Helsinki, Finland). Spontaneous and maximum release were measured in 100 µL of supernatant from wells containing target cells only or target cells with 1% Triton X-100 (Sigma, Saint Louis, MO, USA).

Spontaneous baseline LDH release from target (10^4^ cells well^−1^) was used as controls. The values of the controls were subtracted from the degree of target cell lysis determined according to the equation:

Percent lysis = (measured release − spontaneous target release)/(complete release − spontaneous target release) × 100.

The living cells were observed through Nomarski differential interference contrast optics (DIC). Unless otherwise specified the cytotoxic reactions against tumour cells were carried out at 18 °C for 2 h. This is the optimal temperature for the molecule activity, for this short time the cells neither show any modifications nor do they die.

In addition trypan blue exclusion test was used for dead cells determination by addition of 0.01% trypan blue to the medium. This test was also used to evaluate the cytotoxic activity against tumor cell lines, the dye was added into the reaction mixture after 2 h incubation. To show target cell death following an *in vitro* cytotoxic reaction, the trypan blue was added to the medium 20 min after the mucus were mixed with target cells. Samples of the reaction mixture were smeared on slides and examined under the microscope.

### 4.9. Fractionation of Actinia Equina Mucus

Although ultrafiltration is primarily a separation technique, under some conditions it can be used for the gross fractionation of proteins that differ significantly in size. Briefly, the 10 kDa Nanosep device has been inserted into one of the provided microcentrifuge tubes and 500 µL of mucus sample was added. The filter device was positioned into the centrifuge rotor with a counterbalance with a similar device. After 20 min of centrifugation at 6000× *g* the filtrate was transferred from the bottom receiver to a new tube for storage. The sample with low molecular mass was filtered through the membrane (10 kDa size pores) and collected as down fraction, while the component with higher molecular mass remained above the membrane and was collected as up and stored at −20 °C until the proteic concentration evaluation and the use for the assays.

### 4.10. HPLC Size Exclusion Chromatography

Mucus extract were subjected to size exclusion chromatography using BioSuite 250, 10 μm SEC, 7.5 mm × 300 mm column (Waters, Milford, USA) on a HPLC system (Shimadzu Scientific Instruments, Columbia, MD, USA). The column was washed with Tris buffered saline (TBS) (150 mM NaCl, 10 mM Tris, pH 7.4). 200 μL of each sample were injected into the column which was eluted with TBS at a flow rate of 1 mL/min for 30 min. The chromatogram was recorded with a UV detector at 280 nm (mAU). The collected fractions were concentrated by centrifugation at 500× *g* with micro-concentrators (3 K Omega Centrifugal Devices Nanosep, Pall Corporation, Port Washington, NY, USA), and the final concentrated samples were stored at −80 °C until use.

## 5. Conclusions

Compared with terrestrial ecosystem and organisms, marine ecosystems and biodiversity are largely unexplored and underexploited in terms of potential provision for biomaterials, food, energy, and beneficial services for humans. Here we showed the mucus of the cnidarian sea anemone *A. equina* might represent a novel source of bioactive molecules with potential applicative purposes in drug discovery and biotechnological processes. Further investigations will be required in order to isolate and better characterize the molecular effectors responsible for the observed biological activities of the sea anemone mucus. The search for novel biomolecules deserves the development of appropriate measures to strengthen the focus on untapped source organisms from marine environments.

## References

[1] Connor V.M. (1986). The use of mucous trails by intertidal limpets to enhance food resources. Biol. Bull..

[2] Davies M.S., Jones H.D., Hawkins S.J. (1990). Seasonal variation in the composition of pedal mucus from *Patella vulgata* L.. J. Exp. Mar. Biol. Ecol..

[3] Davis J.M., Viney C. (1998). Water-mucin phases: Conditions for mucus liquid crystallinity. Thermochim. Acta.

[4] Smith A.M., Quick T.J., Peter S.T.R.L. (1999). Differences in the Composition of Adhesive and Non-Adhesive Mucus from the Limpet *Lottia limatula*. Biol. Bull..

[5] Kamino K. (2008). Underwater adhesive of marine organisms as the vital link between biological science and material science. Mar. Biotechnol..

[6] Branch G.M. (1981). The biology of limpets: Physical factors, energy flow and ecological interactions. Oceanogr. Mar. Biol. Ann. Rev..

[7] Martin R., Walther P. (2003). Protective mechanisms against the action of nematocysts in the epidermis of *Cratena peregrina* and *Flabellina affinis* (Gastropoda, Nudibranchia). Zoomorphology.

[8] Baier R.E., Gucinski H., Meenaghan M.A., Wirth J., Glantz P.Q., Glantz P.Q., Leach S.A., Ericson T. (1985). Biophysical studies of mucosal surfaces. Oral Interfacial Reactions of Bone, Soft Tissue and Saliva.

[9] Clare A.S. (1995). Marine natural product antifoulants: Status and potential. Biofouling.

[10] Stabili L., Schirosi R., Licciano M., Giangrande A. (2009). The mucus of *Sabella spallanzanii* (Annelida, Polychaeta): Its involvement in chemical defence and fertilization success. J. Exp. Mar. Biol. Ecol..

[11] Iori D., Forti L., Massamba-N’Siala G., Prevedelli D., Simonini R. (2014). Toxicity of the purple mucus of the polychaete *Halla parthenopeia* (Oenonidae) revealed by a battery of ecotoxicological bioassays. Sci. Mar..

[12] Stabili L., Schirosi R., Licciano M., Giangrande A. (2014). Role of *Myxicola infundibulum* (Polychaeta, Annelida) mucus: From bacterial control to nutritional home site. J. Exp. Mar. Biol. Ecol..

[13] Waite J.H. (1987). Nature’s underwater adhesive specialist. Int. J. Adhes. Adhes..

[14] Derby C.D. (2007). Escape by inking and secreting: Marine molluscs avoid predators through a rich array of chemicals and mechanisms. Biol. Bull..

[15] Mayer A.M., Rodriguez A.D., Taglialatela-Scafati O., Fusetani N. (2013). Marine Pharmacology in 2009–2011: Marine Compounds with Antibacterial, Antidiabetic, Antifungal, Anti-Inflammatory, Antiprotozoal, Antituberculosis, and Antiviral Activities; Affecting the Immune and Nervous Systems, and other Miscellaneous Mechanisms of Action. Mar. Drugs.

[16] Aneiros A., Garateix A. (2004). Bioactive peptides from marine sources: Pharmacological properties and isolation procedures. J. Chromatogr. B Anal. Technol. Biomed. Life Sci..

[17] Otero Gonzalez A.J., Magalhaes B.S., Garcia Villarino M., Lopez Abarrategui C., Sousa D.A., Dias S.C., Franco O.L. (2010). Antimicrobial peptides from marine invertebrates as a new frontier for microbial infection control. FASEB J..

[18] Smith V.J., Desbois A.P., Dyrynda E.A. (2010). Conventional and unconventional antimicrobials from fish, marine invertebrates and micro-algae. Mar. Drugs.

[19] Bavington C.D., Lever R., Mulloy B., Grundy M.M., Page C.P., Richardson N.V., McKenzie J.D. (2004). Anti-adhesive glycoproteins in echinoderm mucus secretions. Comp. Biochem. Physiol. B Biochem. Mol. Biol..

[20] Fountain D.W., Campbell B.A. (1984). A lectin isolated from mucus of *Helix aspersa*. Comp. Biochem. Physiol..

[21] McKenzie J.D., Grigovala I.V. (1996). The echinoderm surface and its role in preventing microfouling. Biofouling.

[22] Canicatti C., D’Ancona G. (1990). Biological protective substances in *Marthasterias glacialis* (Asteroidea) epidermal secretion. J. Zool..

[23] Stabili L., Schirosi R., Di Benedetto A., Merendino A., Villanova L., Giangrande A. (2011). First insights into the biochemistry of *Sabella spallanzanii* (Annelida: Polychaeta) Mucus: A potentially unexplored resource for applicative purposes. J. Mar. Biol. Assoc. UK.

[24] Calow P. (1979). Why some metazoan mucus secretions are more susceptible to microbial attack than others. Am. Nat..

[25] Azam F. (1998). Microbial control of oceanic carbon flux: The plot thickens. Science.

[26] Azam F., Smith D.C., Steward G.F., Hagström A. (1993). Bacteria-organic matter coupling and its significance for oceanic carbon cycling. Microbial Ecol..

[27] Peduzzi P., Herndl G.J. (1991). Mucus trails in the rocky intertidal: A highly active microenvironment. Mar. Ecol. Prog. Ser..

[28] Davies M.S., Hawkins S.J., Jones H.D. (1992). Pedal mucus and its influence on the microbial food supply of two intertidal gastropods, *Patella vulgata* L. and *Littorina littorea* (L.). J. Exp. Mar. Biol. Ecol..

[29] Imrie D.W., Grahame J., Mill P.J., Reid D.G. (1992). The role of pedal mucus in the feeding behaviour of *Littorina littorea* (L.). Proceedings of the 3rd International Symposium on Littorinid Biology.

[30] Wild C., Woyt H., Markus Huettel M. (2005). Influence of coral mucus on nutrient fluxes in carbonate sand. Mar. Ecol. Progr. Ser..

[31] Gunasundari V., Ajith Kumar T.T., Kumaresan S., Balagurunathan R., Balasubramanian T. (2013). Isolation of aliphatic-antibiotic compounds from marine invertebrate, *Heteractis magnifica* “Quoy & Gaimard1833” against captive marine ornamental fish pathogens. Indian J. Geomar. Sci..

[32] Lin X.Y., Ishida M., Nagashima Y., Shiomi K. (1996). A polypeptide toxin in the sea anemone *Actinia equina* homologous with other sea anemone sodium channel toxins: Isolation and amino acid sequence. Toxicon.

[33] Frazão B., Vasconcelos V., Antunes A. (2012). Sea Anemone (Cnidaria, Anthozoa, Actiniaria) Toxins: An Overview. Mar. Drugs.

[34] Flammang P., Santos R., Haesaerts D., Matranga V. (2005). Echinoderm adhesive secretions: From experimental characterization to Evidence-Based Complementary and Alternative Medicine biotechnological applications. Progress in Molecular and Subcellular Biology, Marine Molecular Biotechnology, Echinodermata.

[35] Taylor S.W., Waite J.H., McGrath K., Kaplan D. (1997). Marine adhesives: From molecular dissection to application. Protein-Based Materials.

[36] Hamwood T.E., Cribb B.W., Halliday J.A., Kearn G.C., Whittington I.D. (2002). Preliminary characterization and extraction of anterior adhesive secretion in monogean (plathyelminth) parasites. Folia Parasitol..

[37] DeMoor S., Waite H., Jangoux M., Patrick P. (2003). Characterization of the adhesive from Cuvierian tubules of the sea cucumber *Holothuria forskali* (Echinodermata, Holothuroidea). Mar. Biotechnol..

[38] Pawlicki J.M., Pease L.B., Pierce C.M., Startz T.P., Zhang Y., Smith A.M. (2004). The effect of molluscan glue proteins on gel mechanics. J. Exp. Biol..

[39] Santos R., da Costa G., Franco C., Gomes-Alves P., Flammang P., Coelho A.V. (2009). First insights into the biochemistry of tube foot adhesive from the sea urchin *Paracentrotus lividus* (Echinoidea, Echinodermata). Mar. Biotechnol..

[40] Smith A.M., Morin M.C. (2002). Biochemical differences between trail mucus and adhesive mucus from marsh periwinkles. Biol. Bull..

[41] Flammang P., Walker G. (1997). Measurement of the adhesion of the podia in the asteroid *Asterias rubens* (Echinodermata). J. Mar. Biol. Assoc. UK.

[42] Flammang P.A., van Cauwenberge M.A., Alexandre H., Jangoux M. (1998). A study of temporary adhesion of the podia in the sea star *Asterias rubens* (Echinodermata, Asteroidea) through their footprints. J. Exp. Biol..

[43] Smith A.M., Smith A.M., Callow J.A. (2006). The biochemistry and mechanics of gastropod adhesive gels. Biological Adhesives.

[44] Kamino K., Smith A.M., Callow J.A. (2006). Barnacle underwater attachment. Biological Adhesives.

[45] Jolles P., Jolles J. (1984). What’s new in lysozyme research?. Mol. Cell. Biochem..

[46] Lesser M.P., Stochaj W.R., Tapley D.W., Shick J.M. (1995). Bleaching in coral reef anthozoans: Effects of irradiance, ultraviolet radiation, and temperature on the activities of protective enzymes against active oxygen. Coral Reefs.

[47] Leclerc M., Rinkevich B., Muller W.E.G. (1996). Humoral factors in marine invertebrates. Molecular and Subcellular Biology: Invertebrate Immunology.

[48] Cheng T.C., Rodrick G.E. (1974). Identification and characterization of lysozyme from the hemolymph of the soft-shelled clam, *Mya arenaria*. Biol. Bull..

[49] Maginot N., Samain J.F., Daniel J.Y., Le Coz J.R., Moal J. (1989). Kinetic properties of lysozyme from the digestive glands of *Ruditapes philippinarum*. Oceanis.

[50] Sotelo-Mundo R.R., Islas-Osuna M.A., de-la-Re-Vega E., Hernandez-Lopez J., Vargas-Albores F., Yepiz-Plascencia G. (2003). cDNA cloning of the lysozyme of the white shrimp *Penaeus vannamei*. Fish Shellfish Immunol..

[51] Huag K., Olsen Ø.M., Sandsdalen E., Styrvold O.B. (2004). Antibacterial activities in various tissues of the horse mussel *Modiolus modiolus*. J. Invertebr. Pathol..

[52] Xue O.G., Schey K.L., Volety A.K., Chu F.L.E., La Peyre J.F. (2004). Purification and characterization of lysozyme from plasma of the eastern oyster *Crassostrea virginica*. Comp. Biochem. Physiol. B Biochem. Mol. Biol..

[53] Kristan K., Viero G., Dalla Serra M., Macek P., Anderluh G. (2009). Molecular mechanism of pore formation by actinoporins. Toxicon.

[54] Monastyrnaya M.I., Leychenko E., Isaeva M., Likhatskaya G., Zelepuga E., Kostina E., Trifonov E., Nurminski E., Kozlovskaya E. (2010). Actinoporins from the sea anemones, tropical *Radianthus macrodactylus* and northern *Oulactis orientalis*: Comparative analysis of structure-function relationships. Toxicon.

[55] Slattery M., McClintock J.B., Heine J.N. (1995). Chemical defences in Antarctic soft corals: Evidence for anti-fouling compounds. J. Exp. Mar. Biol. Ecol..

[56] Ibrahim H.R., Aoki T., Pellegrini A. (2002). Strategies for new antimicrobial proteins and peptides: Lysozyme and aprotinin as model molecules. Curr. Pharm. Des..

[57] Niyonsaba F., Ogawa H. (2005). Protective roles of the skin against infection: Implication of naturally occurring human antimicrobial agents β-defensins, cathelicidin LL-37 and lysozyme. J. Dermatol. Sci..

[58] Giraldi T., Ferlan I., Romeo D. (1976). Antitumor activity of equinatoxin. Chem. Biol. Interact..

[59] Mariottini G.L., Robbiano L., Carli A. (1998). Toxicity of *Actinia equina* (Cnidaria: Anthozoa) crude venom on cultured cells. Boll. Soc. Ital. Biol. Sper..

[60] Soletti R.C., de Faria G.P., Vernal J., Terenzi H., Anderluh G., Borges H.L., Moura-Neto V., Gabilan N.H. (2008). Potentiation of anticancer-drug cytotoxicity by sea anemone pore-forming proteins in human glioblastoma cells. Anticancer Drugs.

[61] Soletti R.C., Alves T., Vernal J., Terenzi H., Anderluh G., Borges H.L., Gabilan N.H., Moura-Neto V. (2010). Inhibition of MAPK/ERK, PKC and CaMKII signaling blocks cytolysin-induced human glioma cell death. Anticancer Res..

[62] Parisi M.G., Trapani M.R., Cammarata M. (2014). Granulocytes of sea anemone *Actinia equina* (Linnaeus, 1758) body fluid contain and release cytolysins forming plaques of lysis. ISJ.

[63] Jelic-Mrcelic G., Sliskovic M., Antolic B. (2006). Biofouling communities on test panels coated with TBT and TBT-free copper-based antifouling paints. Biofouling.

[64] Turley P.A., Fenn R.J., Ritter J.C., Callow M.E. (2005). Pyrithiones as antifoulants: Environmental fate and loss of toxicity. Biofouling.

[65] Stupak M.E., Garcia M.T., Perez M.C. (2003). Non-toxic alternative compounds for marine antifouling paints. Int. Biodeter. Biodegr..

[66] Konstantinou I.K., Albanis T.A. (2004). Worldwide occurrence and effects of antifouling paint booster biocides in the aquatic environment: A review. Environ. Int..

[67] Ostroumov S.A. (2008). On the concepts of biochemical ecology and hydrobiology: Ecological chemomediators. Contemp. Probl. Ecol..

[68] Barresi G., di Carlo E., Trapani M.R., Parisi M.G., Chille C., Mule M.F., Cammarata M., Palla F. (2015). Marine organisms as source of bioactive molecules applied in restoration projects. Herit. Sci..

[69] Rosen M.W., Cornford N.E. (1971). Fluid friction of fish slimes. Nature.

[70] Cone R.A., Ogra P.L., Mestecky J., Lamm M.E., Strober W., Bienenstock J., McGhee J.R. (1999). Mucus. Mucosal Immunology.

[71] Withers P.C. (1992). Comparative Animal Physiology.

[72] Kitson R.E., Mellon M.G. (1944). Colorimetric determination of phosphorus as molybdivanadophosphoric acid. Ind. Eng. Chem. Anal..

[73] Quinlan K.P., Desesa M.A. (1955). Spectrophotometric determination of phosphorus as molybdovanadophosphoric acid. Anal. Chem..

[74] Folch J., Less M., Stone Stanley G.H. (1957). A simple method for the isolation and purification of total lipids from animal tissues. J. Biol. Chem..

[75] Zöllner N., Kirsch K. (1962). Determination of the total lipid concentration in serum. Z. Gesamte Exp. Med..

[76] Bradford M. (1976). A rapid and sensitive method for the quantification of microgram quantities of protein utilizing the principle of proteins dye binding. Anal. Biochem..

[77] Dubois M., Gilles K.A., Hamilton J.K., Rebers P.A., Smith F. (1956). Colorimetric method for determination of sugars and related substances. Anal. Chem..

[78] Kennedy J.F., Pagliuca G., Chapling M.F., Kennedy J.F. (1994). Chapter 2. Oligosaccharides. Carbohydrate Analysis A Practical Approach.

[79] Laemmli V.H. (1970). Cleavage of structural proteins during the assembly of the head of bacteriophage T4. Nat. Lond..

[80] Hames B.D., Hames B.D., Rickwood D. (1990). One-dimensional polyacrylamide gel electrophoresis. IRL Gel Electrophoresis of Proteins. A Practical Approach.

[81] Korzeniewski C., Callewaert D.M. (1983). An enzyme-release assay for natural cytotoxicity. J. Immunol. Methods.

